# Impact of Home Telemonitoring and Management Support on Blood Pressure Control in Nondialysis CKD: A Systematic Review and Meta-Analysis

**DOI:** 10.1177/20543581221106248

**Published:** 2022-06-21

**Authors:** Shezel Muneer, Ikechi G. Okpechi, Feng Ye, Deenaz Zaidi, Mohammed M. Tinwala, Laura N. Hamonic, Anukul Ghimire, Naima Sultana, Dan Slabu, Maryam Khan, Branko Braam, Kailash Jindal, Scott Klarenbach, Raj Padwal, Jennifer Ringrose, Nairne Scott-Douglas, Soroush Shojai, Stephanie Thompson, Aminu K. Bello

**Affiliations:** 1 Department of Medicine, University of Alberta, Edmonton, Canada; 2 John W. Scott Health Sciences Library, University of Alberta, Edmonton, Canada; 3 Faculty of Science, University of Alberta, Edmonton, Canada; 4 Division of Nephrology and Immunology, Faculty of Medicine and Dentistry, University of Alberta, Edmonton, Canada

**Keywords:** CKD, hypertension, blood pressure control, telemonitoring, eHealth

## Abstract

**Background:**

Hypertension is a major cause of cardiovascular disease, chronic kidney disease (CKD), and death. Several studies have demonstrated the efficacy of home blood pressure telemonitoring (HBPT) for blood pressure (BP) control and outcomes, but the effects of this intervention remain unclear in patients with CKD.

**Objective:**

To determine the impact of HBPT on cardiovascular–related and kidney disease–related outcomes in patients with CKD.

**Design:**

Systematic review and meta-analysis.

**Setting:**

All studies that met our criteria regardless of country of origin.

**Participants:**

Patients with chronic kidney disease included in studies using HBPT for BP assessment and control.

**Measurements:**

Descriptive and quantitative analysis of our primary and secondary outcomes.

**Methods:**

We searched MEDLINE, Embase, CINAHL Plus, PsycINFO, Cochrane CENTRAL, Web of Science, and gray literature from inception for observational and randomized controlled studies in nondialysis (ND) CKD using HBPT for BP control. We selected studies that used HBPT as intervention (with or without a control arm) for BP control in ND-CKD populations. The primary outcome was change in mean systolic BP (SBP) and mean diastolic BP (DBP).

**Results:**

We selected 7 studies from 1669 articles that were initially identified. Overall, pooled estimates in the mean difference (MD) for SBP and DBP were −8.8 mm Hg; 95% confidence interval (CI): −16.2 to −1.4; *P* = .02 and −2.4 mm Hg; 95% CI: −3.8 to −1.0; *P* < .001, respectively. For studies comparing intervention with usual care (UC), pooled estimate in MD for SBP was −8.0 mm Hg (*P* = .02) with no significant reduction for DBP (−2.6 mm Hg; *P* = .18). In studies without a UC arm, both SBP and DBP were not significantly reduced (*P* > .05). The pooled estimate in MD for estimated glomerular filtration rate showed a significant improvement (5.4 mL/min/1.73 m^2^; *P* < .001).

**Limitations:**

Heterogeneity and few available studies for inclusion limited our ability to identify a robust link between HBPT use and BP and kidney function improvement.

**Conclusion:**

Home blood pressure telemonitoring is associated with mild lowering of BP and moderately improved kidney function in patients with CKD. However, larger studies with improved designs and prolonged interventions are still needed to assess the effects of HBPT on patients’ outcomes.

**PROSPERO registration ID:**

**CRD42020190705**

## Abrégé

**Contexte:**

L’hypertension est une cause majeure de maladie cardiovasculaire, d’insuffisance rénale chronique (IRC) et de mortalité. Plusieurs études ont montré l’efficacité de la télésurveillance de la pression artérielle à domicile (TSPA) pour le contrôle de la pression artérielle (PA) et les évènements cliniques, mais les effets de cette intervention demeurent mal connus chez les patients atteints d’IRC.

**Objectif:**

Évaluer l’effet de la TSPA sur les évènements cardiovasculaires et rénaux chez les patients atteints d’IRC.

**Conception:**

Revue systématique et méta-analyse

**Sources:**

Toutes les études satisfaisant nos critères, peu importe le pays d’origine.

**Sujets:**

Les patients atteints d’IRC inclus dans les études portant sur l’utilisation de la TSPA pour réguler la pression artérielle.

**Mesures:**

Analyse descriptive et quantitative de nos résultats primaires et secondaires.

**Méthodologie:**

Nous avons consulté les bases de données MEDLINE, embase, CINAHL plus, PsycINFO, Cochrane CENTRAL et Web of Science, de même que la littérature grise depuis leur début, à la recherche des études observationnelles contrôlées et randomisées portant sur l’utilisation de la TSPA pour contrôler la PA chez des patients atteints d’IRC non dialysés. Nous avons sélectionné les études (avec ou sans bras témoin) utilisant l’intervention (TSPA pour contrôler la PA) dans des populations de patients atteints d’IRC non dialysés. Le principal critère d’évaluation était un changement de la pression systolique moyenne (PSM) et de la pression diastolique moyenne (PDM).

**Résultats:**

Nous avons retenu sept études parmi les 1 669 articles initialement répertoriés. Dans l’ensemble, les estimations regroupées de la différence moyenne (DM) pour la PSM et la PDM étaient de −8,8 mmHg (IC 95%: −16,2 à −1,4; *P* = 0,02) et de −2,4 mmHg (IC 95%: −3,8 à −1,0; *P* < 0,001) respectivement. Dans les études qui comparaient l’intervention aux soins habituels (SH), les estimations regroupées de la DM s’établissaient à −8,0 mmHg (*P* = 0,02) pour la PSM, sans réduction significative pour la PDM (−2,6 mmHg; *P* = 0,18). Dans les études sans bras SH, aucune réduction significative n’a été observée pour la PSM et la PDM (*P* > 0,05). L’estimation groupée de la DM pour le débit de filtration glomérulaire estimé (DFGe) a montré une amélioration significative (5,4 ml/min/1,73 m^2^; *P* < 0,001).

**Limites:**

Le peu d’études disponibles pour inclusion et leur hétérogénéité limitent notre capacité à établir un lien robuste entre l’utilisation de la TSPA et une amélioration de la PA et de la fonction rénale.

**Conclusion:**

La TSPA est associée à une légère baisse de la PA et à une amélioration modérée de la fonction rénale chez les patients atteints d’IRC. Des études de plus grande envergure, avec des conceptions améliorées et des interventions prolongées, sont nécessaires pour mieux évaluer les effets de la TSPA sur les résultats des patients.

## Introduction

The prevalence of systemic arterial hypertension continues to rise globally, especially in developing countries. The global prevalence of hypertension (defined as systolic blood pressure [SBP] ≥140 mm Hg and/or diastolic blood pressure [DBP] ≥90 mm Hg) in adults was estimated to be 31.1% in 2010, corresponding to 1.38 billion people affected.^1^ Although treatment options are available, blood pressure (BP) control remains suboptimal, particularly in low- and middle-income countries, due to poor adherence, clinical inertia, and organizational failure.^1,2^ A major challenge associated with hypertension care is the proportion of patients able to achieve BP control to target. One large survey involving more than 1.5 million people across 92 countries who were screened for hypertension showed that only 31.7% were controlled to <140/90 mm Hg and 23.3% had untreated or inadequately treated hypertension.^3^ The consequence of poorly controlled hypertension is an increase in target organ damage including cardiovascular (CV) disease, stroke, chronic kidney disease (CKD), and increased mortality.^4-8^

Measurement of BP remains an important aspect of care associated with BP control. Home BP monitoring (HBPM) has been shown to be potentially more reproducible than either office BP monitoring (OBPM) or ambulatory BP monitoring and is more accurate and superior to OBPM in predicting CV events and all-cause mortality.^9,10^ Moreover, as OBPM does not always correctly identify patients with hypertension due to “white-coat” or “masking” effects, HBPM improves BP monitoring and provides more representative BP data and better prediction of outcomes.^11^ The ability to transmit, in real time, data from HBPM device to a care giver improves the chance of better BP control and accelerated delivery of best practice. When combined with decision-making strategies, it can also reduce adverse outcomes associated with hypertension.^12,13^

Home BP telemonitoring (HBPT) is based on the use of electronic automated BP monitors storing BP values obtained at patient’s home and promotes a more effective link between patients and their caregivers.^12,14^ In the Telemonitoring and Self-Management in Hypertensions (TASMINH2) study, participants made medication changes, felt confident about self-monitoring, and felt that their home readings were more valid than office readings taken by their doctor.^15,16^ A following study (TASMINH4) reported that, in comparison with usual care (UC), the mean SBP difference with telemonitoring was −3.5 mm Hg.^17^ Home BP telemonitoring has also been shown to be both cost effective and more effective in achieving BP control than UC.^18^ In addition, when HBPT is combined with additional care (counseling, education, behavioral management, etc), there was a further increased mean change in SBP and DBP as compared with HBPT alone, suggesting that HBPT can be more efficacious when additional support is provided.^18^ A number of other studies have equally demonstrated the efficacy of HBPT on BP control and outcomes.^13,15-17,19,20^ One systematic review that assessed the role of HBPT in patients with CKD and included only 2 studies in quantitative analysis did not find statistically significant changes in SBP, DBP, mean arterial pressure (MAP), or estimated glomerular filtration rate (eGFR) at the end of the studies from baseline values.^21^ This is likely related to the small sample size and limited number of studies included. However, researchers have pointed to the role of out-of-office BP monitoring in patients with CKD, particularly in patients on hemodialysis where self-measured BP but not predialysis or postdialysis BP shows high sensitivity and specificity of >80% to make a diagnosis of hypertension.^22^

## Objectives

We systematically reviewed studies using HBPT in nondialysis (ND) patients with CKD to determine the impact of HBPT and management support on BP control, CV-related and kidney disease–related outcomes in this population.

## Methods

This systematic review and meta-analysis is reported using the established framework of Preferred Reporting Items for Systematic reviews and Meta-Analyses (PRISMA).^23^ The protocol for this review was registered in PROSPERO (CRD42020190705) and has been published.^24^

### Eligibility Criteria

Studies published from inception to June 30, 2020 were included, in all publication languages, if HBPT was used for BP assessment and monitoring irrespective of additional management support by physicians and/or allied health care workers in ND-CKD adults (≥18 years of age). We accepted the definition of CKD as used by the authors. We also included observational studies, randomized controlled trials (RCTs), and published abstracts that evaluated telemonitoring for BP control and reported at least 1 outcome. Studies comparing HBPT with standardized care and other interventions, such as counseling and education around BP management, use of other BP measuring devices, etc, were included, as well as HBPT studies in ND-CKD with no comparators. Studies were excluded if other forms of eHealth (eg, telemedicine, short message service [SMS]) were used for BP assessment, if a specific pharmacological management was utilized for BP control, if patients were receiving kidney replacement therapy (KRT), and if BP change was not reported or cannot be deduced. We also excluded review articles, editorials, letters to the editor, commentaries, case studies, case reports, images, and studies from which we were unable to gather relevant data even after attempts to obtain these from the authors. We defined HBPT as the use of an electronic automated BP monitor storing BP readings obtained at home and transmitted to a remote computer through a wired or wireless telephone line, a modem, or an Internet connection.^25^

### Outcomes

The primary outcome was defined as any change in mean SBP, mean DBP, MAP, and/or proportion of participants with controlled BP as defined by the study investigators. Secondary outcomes included clinical and biochemical factors related to kidney disease (eg changes in eGFR, proteinuria, or requirement for KRT), hospitalizations, incident fatal and nonfatal CV events, all-cause mortality, cost effectiveness, patient-reported outcome measures, and patient-reported experience measures.

### Search Strategy for Identifying Relevant Studies

We searched MEDLINE (Ovid), Embase (Ovid), CINAHL Plus with Full Text (EBSCO), PsycINFO (Ovid), Cochrane CENTRAL (Cochrane Library), Web of Science, and Dissertations and Theses Global (ProQuest). Searches employed both controlled vocabularies, such as Medical Subject Headings (MeSH), and keywords representing concepts such as: (hypertension or blood pressure) AND (chronic kidney disease or chronic renal insufficiency) AND (telemedicine or virtual monitoring). The search was conducted by our medical librarian (L.H.) and the strategy was reviewed for completeness and accuracy using Peer Review of Electronic Search Strategies (PRESS)^26^ by a second research librarian. No limitations or filters were used, and search strategies were adapted for each database (Supplementary Table S1).

## Study Selection

**Figure 1.**
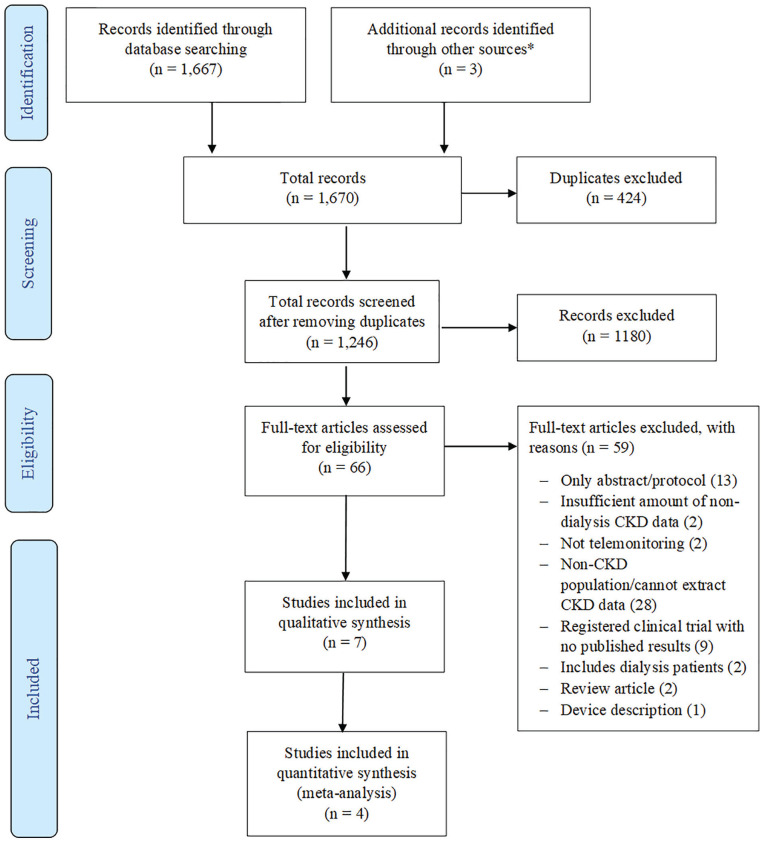
Study selection process. *Source.* Other sources—references from other articles. *Note*. CKD = chronic kidney disease; qualitative synthesis = descriptive and thematic analysis only; quantitative synthesis = analysis involving pooled data (meta-analysis).

Identified studies were added to the Covidence database (https://www.covidence.org/) and 2 reviewers (S.M. and M.T.) independently screened the titles and abstracts of the records retrieved by database searches. Subsequently, full texts of all potentially eligible articles were obtained and further assessed for final inclusion (Figure 1—PRISMA flow chart). Disagreements were resolved by a third reviewer (I.G.O.).

### Data Extraction

Data were extracted onto a predeveloped data capture sheet and reviewed by 2 investigators (D.Z. and I.G.O.) for accuracy and completeness. The following data elements were collected for each study as detailed below:

*Study characteristics*: Publication year, country, study design, sample size, study length, intervention length, management support type, and type of HBPT device used.
*Participant characteristics*: Total number of participants, total number and percent of intervention and control patients (where applicable), CKD stage or eGFR value, and comorbidities (eg, number of diabetic patients). We also collected data related to participants’ clinical and biochemical variables including antihypertensive medications, mean SBP, DBP, creatinine, and eGFR levels.

### Risk of Bias in Individual Studies

We adapted and used the 9-item tool developed by Hoy et al^27^ and used it to assess the methodological quality of included studies (Supplementary Table S2). Studies were classified according to their overall score as high (1-3), medium (4-6), or low (7-9) quality. Three reviewers (M.T., D.Z., I.G.O.) independently assessed the quality of the studies and we assessed interrater agreement for study inclusion using the kappa (κ) coefficient.^28^

## Data Synthesis and Analysis

Results were pooled according to the study designs. In before-after studies (or HBPT arm-only studies), the mean difference (MD) was calculated as the follow-up mean minus the baseline mean (change from baseline). If the change from baseline SD was not available, then a within-group conservative correlation of 0.5 was assumed, as previously reported.^29,30^ In studies with control groups, the MD was calculated as the change from baseline in the HBPT group minus the change from baseline in the UC group. Missing SDs were calculated using 95% confidence intervals (CIs) or imputed using the interquartile range (IQR) when available.^31,32^ If no data on spread were reported, then missing SDs were imputed using the arithmetic mean of the SDs from the same study designs.^31,32^ A random-effects model was used to pool the MDs.^33^ Statistical heterogeneity was tested using the χ^2^ test (considering a value of *P* <.1 to indicate heterogeneity) and quantified using the *I*^2^ statistic (*I*^2^ values of <25%, 25-50%, and >50% represent low, medium, and high heterogeneity, respectively).^29^ Due to small numbers of contributing studies (<10 within study design), there was insufficient power to assess publication bias^34^ and examine subgroups. Sensitivity analysis was performed using follow-up mean values only to calculate MD in the studies with control groups for meta-analysis. Data were analyzed using Stata, Version 15.1 (StataCorp LP, College Station, TX).

## Patients and Public Involvement

Patients and the public were not involved.

## Results

### Baseline Demographic, Clinical, and Laboratory Features of Included Studies

Our search identified 1669 articles; after removing duplicates, we screened 1245 articles by titles and abstract. Following this, we reviewed the full texts of 66 articles from which 7 articles^35-41^ were selected for inclusion (Figure 1). The included studies were conducted across 6 countries (the United States,^35,39^ Belgium,^36^ Taiwan,^37^ Japan,^38^ Canada,^40^ and the United Kingdom^41^), comprising 3 RCTs^35,37,39^ and 4 observational studies (2 were described as pilot implementation studies) with an overall sample size of 821 participants. The mean age of participants ranged from 58 to 75.3 years; 5 of the studies^37-41^ reported more male participants (55%-98.5%), and 3 studies^35,39,40^ reported more white participants. Physicians, general practitioners, and/or interdisciplinary teams (physicians, nurses, pharmacists, etc) provided management support in 5 (71.4%) of the studies (Table 1). Quality assessment of the studies (interrater agreement: 57.1%) revealed 5 of the studies (71.4%) to be of moderate quality,^35-37,40,41^ with 1 low-quality^38^ and 1 high-quality^39^ study. Baseline clinical and laboratory features are summarized in Table 2. Mean baseline SBP and DBP in the studies ranged from 127.8 to 152.5 mm Hg and 70.3 to 82 mm Hg, respectively. Only 2 studies^35,40^ reported the total number of medications taken by patients, while 5 studies^35-37,39,41^ reported baseline data on eGFR (range: 25.0-39.4 mL/min/1.73 m^2^).

**Table 1.** Baseline Demographic Features of Studies Included in This Review.

First author (Ref.)	Publication year	Study design	Country	Study quality	Sample size	Study duration (months)	Ethnicity (%)	Age (years)	Sex (male; %)	Patients with DM (%)	Intervention length	CKD stages included	Management support	HBPT device used
Rifkin et al^35^	2013	RCT	United States	Moderate	43	6	HBPT: black—25% UC: black—27%	HBPT: 68.5 ± 7.5 UC: 67.9 ± 8.4	NR	NR	6 months	3-5	Physicians or study pharmacist called to discuss BP readings, provide counseling, or adjust medications as indicated (assistance of a nurse and an IT support team)	An automatic oscillometric BP unit and the home health hub which receives BP and pulse data via Bluetooth from the BP unit, and relays that data through the Internet to a secure Web site
Daelemans et al^36^	2014	Pilot	Belgium	Moderate	15	4.7	NR	75	NR	NR	10 days	NR	General practitioner in consultation with the nephrologist	An ESH-validated, automatic upper arm BP monitor paired with a Bluetooth-enabled mobile phone
Lin et al^37^	2014	RCT	Taiwan	Moderate	36	6	NR	HBPT: 65.7 ± 11.4 UC: 69.8 ± 16.4	61	NR	6 months	3-5	Physicians verified patient BPs in their order entry system weekly, and more frequently if required as per the study group.	Cloud-based manometers integrated with physician order entry systems
Sawai et al^38^	2015	OBS	Japan	Low	54	23	NR	70.4 ± 11.9	81.4	NR	23 months	NR	None	Device able to measure and transmit BP, pulse, and room temperature
Ishani et al^39^	2016	RCT	United States	High	601	12 for 91.0% 24 for 3.8%	HBPT: white—98.7% UC: white—93.3%	HBPT: 75.3 ± 8.1 UC: 74.3 ± 8.1	98.5	42.6	1 year	3-5	Interdisciplinary team (nephrologist, nurse practitioner, nurses, clinical pharmacy specialist, psychologist, social worker, telehealth care technician, and dietician)	An HBPT device and all the peripherals (BP cuff, scale, glucometer, pulse oximeter, stethoscope, and web camera)
Ong et al^40^	2016	Pilot	Canada	Moderate	47	NR	White—70% Asian—27% Black—3%	59.4 ± 14	55	15	6 months	4 and 5	A multidisciplinary team determined by medical severity (eg, nurse and/or pharmacist only or nurse, pharmacist, and physician). The threshold values for critical alerts were established for each patient by their physicians and could be changed at any time.	A smartphone with a preinstalled self-management application and a Bluetooth-enabled home BP monitoring device which was paired to the smartphone for seamless transfer of BP readings
Warner et al^41^	2018	OBS	United Kingdom	Moderate	25	3	NR	58 ± 11	84	20	3 months	3-5	None	An “off-the-shelf” Bluetooth-enabled BP monitor and a tablet computer with custom-developed software

*Note*. RCT = randomized controlled trial; OBS = observational study; UC = usual care; HBPT = home blood pressure telemonitoring; DM = diabetes mellitus; BP = blood pressure; CKD = chronic kidney disease; NR = not reported; IT = information technology; ESH = European Society of Hypertension.

**Table 2.** Baseline Clinical and Laboratory Features Reported From Studies Included in This Review.

First author (Ref.)	Uncontrolled hypertension (%)^a^	SBP	DBP	No. of BP transmissions per month	No. of medications	No. of antihypertensives	eGFR (mL/min/1.73 m^2^)
Rifkin et al^35^	100	HBPT: 149 ± 16.2 UC: 147 ± 8.6	HBPT: 78 ± 12.4 UC: 81 ± 11.2	29 (IQR 22-53)	HBPT: 11.2 ± 4.1 UC: 11.3 ± 4.6	HBPT: 3.9 ± 1.4 UC: 3.9 ± 1.4	HBPT: 37.3 ± 14.2 UC: 39.4 ± 10.6
Daelemans et al^36^	NR	151	74	2 measurements 3 times/d	NR	NR	28
Lin et al^37^	NR	HBPT (am): 137.7 ± 17.3 HBPT (pm): 135.2 ± 14.5 UC (am): 129.7 ± 9.4 UC (pm): 134.8 ± 13.6	HBPT (am): 80.0 ± 6.6 HBPT (pm): 77.0 ± 6.5 UC (am): 76.0 ± 9.0 UC (pm): 75.4 ± 7.6	NR	NR	NR	HBPT: 29.8 ± 17.1 UC: 25.0 ± 14.7
Sawai et al^38^	NR	127.8 ± 14.4	74.3±10.2	NR	NR	NR	NR
Ishani et al^39^	Total: 32.1% HBPT: 33.5% UC: 28.0%	Total: 133.1 ± 19.7 HBPT: 134.3 ± 19.9 UC: 129.6 ± 18.8	Total: 70.9 ± 11.9 HBPT: 71.0 ± 12.0 UC: 70.3 ± 11.4	14.9 ± 10.9	NR	NR	HBPT: 37 ± 9 UC: 38 ± 8
Ong et al^40^	36	130.6 ± 17.2	78.9 ± 10.9	NR	9.5 ± 4.2	NR	NR
Warner et al^41^	NR	152.5 ± 16.2	82	54 readings in a 90-day period)	NR	NR	36 ±13.3

*Note*. SBP = systolic blood pressure; DBP = diastolic blood pressure; BP = blood pressure; eGFR = estimated glomerular filtration rate; HBPT = home blood pressure telemonitoring; UC = usual care; am = morning; pm = nighttime; NR = not reported; IQR = interquartile range.

a Uncontrolled hypertension (>140/90 mm Hg).

### Summary of Reported Outcomes and the Effects of Interventions From Individual Studies

Reported outcomes and effects of interventions from each study are summarized in Table 3. Although all the studies reported baseline BP, only 2 studies^36,37^ focused on improvement of BP throughout the study period, 3 studies^35,40,41^ focused on the acceptability of HBPT to patients with CKD, and 1 study^38^ focused on the effect of seasonal temperature variation on HBPT. One study^39^ that assessed a primary composite endpoint of death, hospitalization, emergency department visits, and admission to a skilled nursing facility failed to show that HBPT yields significantly better outcomes than UC.

**Table 3.** Change in BP, Summary of Reported Outcomes, and Summary of the Effects of Interventions for Studies Included in This Review.

First author (Ref.)	Summary of outcomes assessed/reported	Summary of the effects of interventions
Rifkin et al^35^	Primary endpoints: Improved data exchange and device acceptability. Secondary endpoints: BP change, SBP, DBP, MAP, creatinine, eGFR, total number of medications, number of BP medications, and medication adherence were measured at the start and end of the study.	Average start-of-study BP was 147/78 mm Hg. Those in the intervention arm had a median of 29 (IQR: 22, 53) transmitted BP readings per month, with 78% continuing to use the device regularly, whereas only 20% of those in the UC group brought readings to in-person visits. Both groups had significant improvement in SBP (*P* < .05), which fell a median of 13 mm Hg in monitored participants compared with 8.5 mm Hg in UC participants (*P* = .31). Summary: This low-cost wireless monitoring strategy led to greater sharing of data between patients and clinics and produced a trend toward improved BP control over UC at 6 months.
Daelemans et al^36^	1. Improvement of BP control in patients with CKD 2. Is HBPT an added value for both patients and general practitioners?	Most general practitioners consider HBPT to add value when additional support is provided by the care program promotor (patient education and reporting) and when feedback from a nephrologist is available. Patients who use HBPT experience better follow-up (faster evaluation of results and modification of medication regimens by general practitioners). Summary: HBPT results in better and faster BP control. HBPT adds value, but support of both patients and general practitioners is essential.
Lin et al^37^	A composite endpoint was defined as the changes in each patient’s BP as well as assessments of renal function, including changes in eGFR, creatinine, and urine protein excretion.	Nighttime SBP and DBP were significantly lower in the HBPT group compared with the UC group. Serum creatinine level in the study group improved significantly compared with the control group after the end of month 6 (2.83 ± 2.0 vs 4.38 ± 3.0, *P* = .018). Proteinuria improved nonsignificantly in month 6 in the study group compared with the control group (1.05 ± 0.9 vs 1.90 ± 1.3, *P* = .09). Both SBP and DBP during the nighttime hours improved significantly in the study group compared with baseline. Summary: Regularly monitoring BP by integrating cloud-based manometers appears to result in a significant decrease in creatinine and improvement in nighttime BP control.
Sawai et al^38^	Effect of HBPT on seasonal variation in home BP measurements.	Average SBP and DBP were highest in winter and lowest in summer (*P* < .01); room temperature was also significantly different in all 4 seasons. In logistic regression analysis, only eGFR was significantly correlated with seasonal home BP variation, even after adjustments for age, sex, BMI, DM, room temperature, and baseline SBP (*P* < .05). Summary: HBPT revealed seasonal home BP variation correlating with eGFR. Careful home BP monitoring is important for optimal BP control, especially among patients with CKD.
Ishani et al^39^	Primary composite outcome: Death, hospitalization, emergency department visits, and admission to a skilled nursing facility. Secondary outcomes: Each component of the primary outcome plus incidence of ESKD, second hospitalization, second emergency department visit, number of days of first hospital visit, number of days of all hospitalizations, number of hospitalizations within a year. Intermediate outcomes: SBP ≤140, LDL-C ≤100 mg/dL, HbA1c ≤8%, cessation of smoking.	One year after randomization, 46.2% of patients in the HBPT group versus 46.7% of patients in the UC group experienced the primary composite outcome (HR: 0.98; 95% CI: 0.75-1.29; *P* = .9). No difference was observed between groups for any component of the primary outcome: all-cause mortality (HR: 1.46; 95% CI: 0.42-5.11), hospitalization (HR: 1.15; 95% CI: 0.80-1.63), emergency department visits (HR: 0.92; 95% CI: 0.68-1.24), or nursing home admission (HR: 3.07; 95% CI: 0.71-13.24). Summary: HBPT by an interdisciplinary team is a feasible care delivery strategy in patients with CKD. There is no statistically significant evidence that this intervention yields superior health outcomes compared with UC.
Ong et al^40^	Acceptability of HBPT devices in managing patients with advanced CKD and changes in several clinical parameters (BP, medications, CKD-related symptoms, CKD-specific laboratory tests).	User adherence was high (>80% performed ≥80% of recommended assessments) and sustained. Mean reductions in home BP readings between baseline and exit were statistically significant (SBP: −3.4 mm Hg, 95% CI: −5.0 to −1.8; DBP: −2.1 mm Hg, 95% CI: −2.9 to −1.2); 27% of patients with normal clinic BP readings had newly identified masked hypertension. Among the 127 medication discrepancies identified, 59% were medication errors requiring intervention to prevent harm. In exit interviews, patients indicated feeling more confident and in control of their conditions; clinicians perceived patients to be better informed and more engaged. Summary: Integrating a smartphone-based self-management system into UC of patients with advanced CKD proved feasible and acceptable, and appeared to be clinically useful. The results provide a strong rationale for an RCT.
Warner et al^41^	Acceptability of HBPT to patients with CKD and whether patients would provide sufficient BP readings to assess variability and guide treatment.	User adherence was high: 13/25 (52%) participants provided >90% of the expected data, and 18/25 (72%) provided >80% of the expected data. The usability of the telemonitoring system was rated highly, with mean scores of 84.9/100 (SE 2.8) after 30 days and 84.2/100 (SE 4.1) after 90 days. The coefficient of variation for the variability of SBP telemonitoring was 9.4% (95% CI: 7.8-10.9) compared with 7.9% (95% CI: 6.4-9.5) for the BPro device (*P* = .05). Summary: Telemonitoring is acceptable for patients with CKD and provides sufficient data to inform titration of antihypertensive therapies in either an RCT setting (comparing BP among different targets) or routine clinical practice. Such methods could be employed in both scenarios and reduce costs currently associated with such activities.

*Note*. SBP = systolic blood pressure; DBP = diastolic blood pressure; MAP = mean arterial pressure; HBPT = home blood pressure telemonitoring; UC = usual care; CKD = chronic kidney disease; ESKD = end-stage kidney disease; eGFR = estimated glomerular filtration rate; LDL-C = low density lipoprotein cholesterol; HR = hazard ratio; CI = confidence interval; IQR = interquartile range; RCT = randomized controlled trial; BP = blood pressure; BMI = body mass index; DM = diabetes mellitus.

## Pooled Summary of Study Outcomes

### BP Change

**Figure 2.**
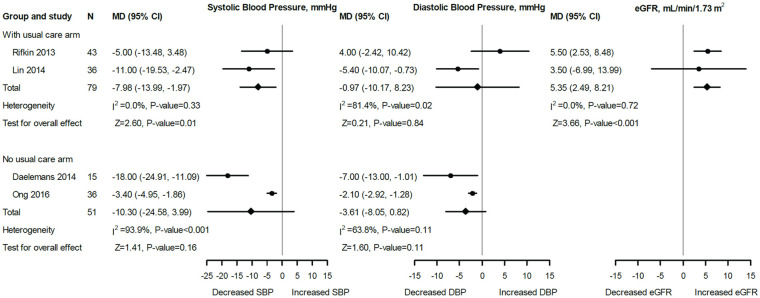
Forest plot and meta-analysis for the effect of HBPT on SBP, DBP, and eGFR after 6 months of follow-up. *Note*. UC arm represents studies that included both HBPT and UC groups. The MD was defined as the difference between HBPT and UC in the mean changes from baseline (ie, follow-up minus baseline values). No UC arm represents studies that included a HBPT arm only. The MD was calculated as the follow-up mean minus the baseline mean. SBP = systolic blood pressure; DBP = diastolic blood pressure; eGFR = estimated glomerular filtration rate; MD = mean difference; CI = confidence interval; HBPT = home blood pressure telemonitoring; UC = usual care.

**Figure 3.**
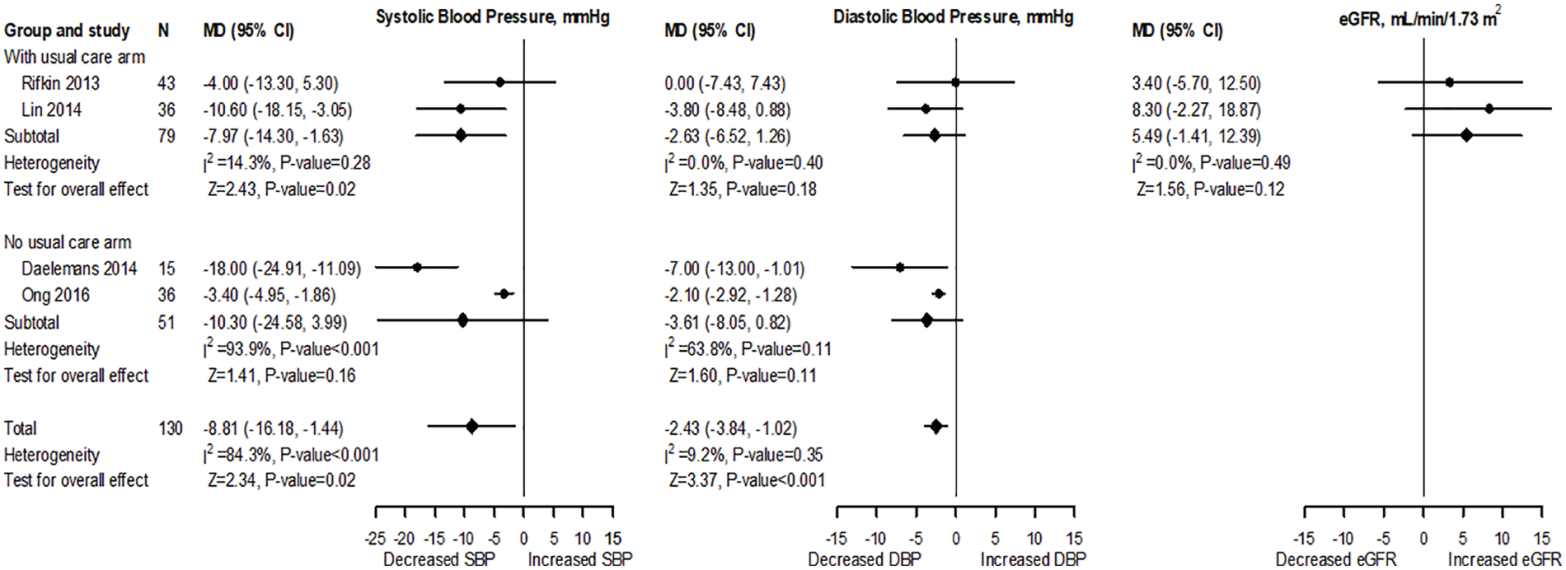
Forest plot and meta-analysis for the effect of HBPT on SBP, DBP, and eGFR using 6-month follow-up mean values (sensitivity analysis). *Note*. UC arm represents studies that included both HBPT and UC groups, and the MD was defined as the difference of the follow-up mean values. No UC arm represents studies that included a HBPT arm only, and the MD was calculated as the follow-up mean minus the baseline mean. SBP = systolic blood pressure; DBP = diastolic blood pressure; eGFR = estimated glomerular filtration rate; MD = mean difference; CI = confidence interval; HBPT = home blood pressure telemonitoring; UC = usual care.

Four studies^35-37,40^ were included in meta-analysis for BP. Two studies^35,37^ with a UC arm assessed SBP and DBP changes after 6 months of follow-up in 79 participants. The baseline SBP and DBP measurements of the 2 studies were not significantly different (Supplementary Figure S1). The pooled data, however, showed a significantly lower SBP (MD: −7.98 mm Hg; 95% CI: −13.99 to −1.97; *I*^2^ = 0.0%; *P* = .01) and a nonsignificant difference in DBP (MD: −0.97 mm Hg; 95% CI: −10.17 to 8.23; *I*^2^ = 81.4%; *P* = .84; Figure 2) between HBPT and UC groups. Two other studies^36,40^ without a UC arm assessed BP changes after 6 months of follow-up in 51 patients. The pooled results showed no significant difference in both SBP (MD: −10.3 mm Hg; 95% CI: −24.58 to 3.99; *I*^2^ = 93.9%; *P* = .16) and DBP (MD: −3.61 mm Hg; 95% CI: −8.05 to 0.82; *I*^2^ = 63.8%; *P* = .11; Figure 2). Changes in SBP after 3 months of intervention were reported by 2 of the studies (without a UC arm in 53 participants)^35,41^ with pooled estimate showing a significant MD (−11.11 mm Hg; 95% CI: −15.36 to −6.85; *I*^2^ = 0.0%; *P* < .001; Supplementary Figure S2). The summary estimates remained essentially unchanged in sensitivity analyses using all follow-up data; however, MD in overall SBP (n = 130; MD: −8.8 mm Hg; *P* = .02) and DBP (n = 130, MD: −2.4 mm Hg; *P* < .001) were significantly reduced (Figure 3).

### eGFR Change

Two studies^35,37^ with a UC arm and baseline GFR values that were not significantly different (Supplementary Figure S1) evaluated changes of eGFR after 6 months of follow-up in 79 participants. The pooled results showed a significant difference (MD: 5.35 mL/min/1.73 m^2^; 95% CI: 2.49-8.21; *I*^2^ = 0.0%; *P* < .001; Figure 2). However, sensitivity analysis using all the follow-up measurements showed that there was a nonsignificant difference (MD: 5.49 mL/min/1.73 m^2^; 95% CI: −1.41 to 12.39; *I*^2^ = 0.0%; *P* = .12; Figure 3).

### Antihypertensive Medication Change

Only 1 study^35^ reported the changes in number of antihypertensive medications after 6 months of follow-up (MD: 0.10; 95% CI: −1.57 to 1.77).

## Discussion

Systemic arterial hypertension is a public health problem that continues to increase globally and remains a major cause of CV disease, CKD, and death.^1,4,5,8^ The aim of this study was to assess the impact of HBPT in patients with CKD and our results have demonstrated a statistically significant impact of HBPT on overall SBP and DBP reduction as well as some improvement of kidney function (eGFR); however, no significant changes in DBP (within 3 months and when studies were separated into groups—with UC vs without UC) or number of antihypertensive medications required by patients with CKD were observed. Qualitative analysis of our study showed that HBPT was acceptable to patients with CKD with no significant effect on patient hospitalization, readmission, and mortality.

The MD for BP was only significantly reduced for SBP in studies comparing HBPT and UC (−7.9 mm Hg; *P* = .02), whereas the MD was not significantly reduced for DBP (−2.6 mm Hg; *P* = .18) and for both SBP and DBP in studies without UC (−10.3 mm Hg; *P* = .16 and −3.61 mm Hg; *P* = .11), respectively (Figure 3). Luo et al^21^ had also assessed the effects of telehealth on BP management in ND-CKD (stages 3-5 only) but did not identify significant reductions in SBP and DBP. They suggested 2 main reasons for this: (1) lack of significance due to statistically low sample size of telehealth studies in patients with CKD and improper study designs of those studies and (2) telehealth for BP management in CKD may be clinically ineffective as it fails to meet the demands of clinical practice.^21^ Based on the results of our data, we think both reasons could apply as the increased sampling in our study was able to detect a significant reduction in SBP in studies comparing HBPT with UC and that the studies we included used different numbers of BP transmission, duration of telemonitoring, and management support for BP evaluation and control in their patients (Tables 2 and 3).

Other studies have suggested that a clinically significant BP-lowering effect of home (self) BP monitoring is proportional to the intensity of co-interventions administered as well as use of a multidisciplinary team.^21,35,42^ One systematic review that included 25 RCTs reported that effect of self-monitoring of BP was strongly influenced by the intensity of co-intervention ranging from no effect with self-monitoring alone (−1.0 mm Hg; 95% CI: −3.3 to 1.2) to a reduction when monitoring was combined with intensive support (6.1 mm Hg; 95% CI: −9.0 to −3.2).^42^ Also, a cluster randomized study that included of a pharmacist for management support in the HBPT group reported significant BP reductions at 12 months that persisted during 6 months of postintervention follow-up.^19^ How management support was utilized in the included studies may have influenced the extent of pooled BP reduction observed in our study. Although modest, with most BP reductions not statistically significant, they are, however, clinically important reductions as several studies have already shown the benefits of even small reductions in SBP and DBP for primary prevention with up to 10% reduction in CV mortality and up to 20% to 30% reduction in major CV events.^43-45^ Additional factors that influence changes in BP when using HBPT have been reported to include the duration of the intervention, the frequency of remote transmission of BP data, additional interventions, and the intervention pathway (mobile phone/web, telephone-linked computer system, or telephone) on HBPT.^46^ Although the differences in these factors across studies we included could have contributed to the measure of change in both SBP and DBP in our pooled analysis, the availability of only a few studies using HBPT in patients with CKD may have had more significant effects on our findings.

Hypertension is associated with more rapid decline of eGFR and there is evidence that BP treatment in patients with CKD may attenuate the decline in eGFR.^47,48^ Two of the studies included in this meta-analysis^35,37^ reported nonsignificantly improved eGFR in the intervention group at 6 months. Rifkin et al showed that the eGFR in the HBPT group was maintained at the end of the study (0.6 mm Hg; IQR: −3.4 to 1.8) with a decline in the UC group (−3.69 mm Hg; IQR: −6.2 to 0; *P* = .14). Although the pooled effect of BP reduction between HBPT and UC on eGFR from both studies demonstrated a significant increase (5.35 ml/min/1.73 m^2^; 95% CI: 2.49-8.21; *P* =<.001), sensitivity analyses using all follow-up data showed a nonsignificant increase in eGFR (5.66 ml/min/1.73 m^2^; 95% CI: −1.51 to 12.83; *P* = .12) suggesting the initial analysis to be an overestimation of the true effects of HBPT on eGFR (Figures 2 and 3). Unfortunately, the lack of clinical trials that have evaluated the effects of HBPT for BP control on kidney function could not allow for a robust assessment of the effects of using this intervention on kidney function.^15,17,19,49^ We think that the lack of clinical trials may be related to the high cost associated with using HBPT, especially given the high cost associated with CKD treatment. Stoddart et al^50^ found HBPT costs to be significantly higher than UC (*P* < .001) with the increased costs related to telemonitoring services, patient training, and additional general practitioner and nurse consultations. Despite the associated cost, other studies have shown HBPT to be more cost-effective than home monitoring alone or UC.^51^ Another reason for the lack of clinical trials might also be the assumption that eHealth interventions in patients with CKD lack efficacy. A large systematic review designed to evaluate the benefits and harms of using eHealth interventions in people with CKD found eHealth interventions to only improve the management of dietary sodium intake and fluid management (out of 98 outcomes categorized into 9 domains).^52^ This was reported to be due to the low quality of studies with uncertain effects and due to methodological limitations and heterogeneity of eHealth modalities and intervention types. More RCTs are clearly needed to assess the role of HBPT on kidney function both in patients with and without CKD.

Finally, our study showed an increased MD in number of antihypertensives prescribed to patients at 6 months from baseline, and qualitative analysis showed that patients found the intervention to be acceptable with no significant effects of HBPT on patient hospitalization, readmission, and mortality. The increased number of antihypertensives highlights one of the advantages of using HBPT for BP assessments as dose and/or number of medications can be adjusted by the intervention team to improve BP control as well as limit medications side effects.^40^ This is corroborated by other studies^17,19^ that have shown that intervention patients had greater antihypertensive medication intensification and better self-reported adherence to antihypertensive medications than UC patients. Some studies have also shown improved adherence to lifestyle measures (eg, salt restriction, increased physical activity, and reduced consumption of alcohol), as an intervention to improve BP in the intervention group than the UC group, suggesting the additive effects of co-interventions in BP reduction.^19^ The impact of HBPT on patient’s attitude was not consistently reported in this study as only 3 of the included studies assessed the acceptability of HBPT.^35,40,41^ However, in all 3 studies, patients found this method of BP assessment to be acceptable. Although the cost of equipment and nonadherence to BP measurements could likely contribute to the acceptability and persistence of telemonitoring techniques, Warner et al^41^ showed high levels of acceptability of HBPT technique in their study at 3 months as they reported 92% acceptability for participants providing >65% BP readings. Adherence was also high at 1 year in the TASMINH4 study, suggesting high acceptability of the technique. Similarly, in the TASMINH2 study, patients found the intervention to be acceptable which increased their confidence in taking control of their own care by self-titrating medications when required.^16^ Further, age and frailty could influence the associations of HBPT and related outcomes. A study by Takahashi et al^53^ that assessed changes in hospitalizations and emergency visits among older adults (patients’ mean age 80.3 ± 8.2 years, 20.5% with CKD) showed that telemonitoring did not lead to reduced hospitalizations or ED visits. These findings as reported by Takahashi et al could be explained by the select nature of their cohort and baseline comorbidity burden.^53^ Thus, the results of ongoing larger pragmatic RCTs utilizing HBPT in patients with CKD, such as the eNephro study^54^ (NCT02082093) and one by our group, the telemonitoring of hypertensive patients with CKD (Telemonitoring for Improved Kidney Outcomes [TIKO]) study (NCT04098354), are likely to improve our understanding of the efficacy, cost utility, and acceptability of HBPT interventions in patients with CKD.

There were some limitations in our study including heterogeneity across studies which may be explained by differences in clinical settings, telemonitoring technologies, timing of self-monitoring, number of antihypertensive medications, number of BP readings/transmissions, differences in BP targets, and the features of the comparative group. Also, due to heterogeneity with CKD definitions and lack of eGFR stratification, we were unable to conduct subgroup analyses by stage of CKD. One study^38^ assessed the effect of seasonal temperature variation on BP values without providing further patient details and was not included in the meta-analysis. Finally, the low sample size of included studies (eg, the sample size of all included RCTs was only 683 individuals)^35,37,39^ may have contributed to limiting the magnitude of the observed effectiveness of HBPT on outcomes and could be due to low availability of studies using telemonitoring technologies for BP management in CKD populations. Despite these limitations, as we have shown a significant effect of HBPT on SBP and kidney function as well as an increased acceptance of this technology, this may support limited use of HBPT in patients with CKD. Although this study exposes the scant availability of properly designed trials with adequate sample size to address the issue of efficacy of HBPT in patients with CKD, it however reveals the need for more RCTs evaluating the effects of HBPT on kidney function. One such RCT is the ongoing Canadian study—TIKO study (www.clinicaltrials.gov registration number: NCT04098354).^55^ This study will assess mean change in SBP, kidney outcomes, and acceptability of HBPT at 12 months, in patients with CKD randomized to HBPT + UC versus HBPT + case manager.^55^ Furthermore, through the delivery of the knowledge translation process developed for this study (Supplementary Appendix), the benefits of HBPT can be extended and broadly implemented by other knowledge users.

## Conclusion

This systematic review and meta-analysis show that HBPT is associated with mild reductions of SBP and moderately improved kidney function in patients with CKD, likely modulated by HBPT effects on SBP. Although our study shows that this intervention is acceptable to patients with CKD, our results should be taken cautiously given the identified limitations including small sample size of included studies and differences in HBPT technologies utilized across different studies. We hope that future studies as well as ongoing RCTs with larger sample sizes and prolonged intervention duration may be able to provide more robust evidence of the usefulness of HBPT on BP, kidney, and CV outcomes in patients with CKD.

## Supplemental Material

sj-pdf-1-cjk-10.1177_20543581221106248 – Supplemental material for Impact of Home Telemonitoring and Management Support on Blood Pressure Control in Nondialysis CKD: A Systematic Review and Meta-Analysis Click here for additional data file. Supplemental material, sj-pdf-1-cjk-10.1177_20543581221106248 for Impact of Home Telemonitoring and Management Support on Blood Pressure Control in Nondialysis CKD: A Systematic Review and Meta-Analysis by Shezel Muneer, Ikechi G. Okpechi, Feng Ye, Deenaz Zaidi, Mohammed M. Tinwala, Laura N. Hamonic, Anukul Ghimire, Naima Sultana, Dan Slabu, Maryam Khan, Branko Braam, Kailash Jindal, Scott Klarenbach, Raj Padwal, Jennifer Ringrose, Nairne Scott-Douglas, Soroush Shojai, Stephanie Thompson and Aminu K. Bello in Canadian Journal of Kidney Health and Disease
