# Supplementary material for: Impact of Home Telemonitoring and Management Support on Blood Pressure Control in Nondialysis CKD: A Systematic Review and Meta-Analysis
Source: Can J Kidney Health Dis. 2022 Jun 21;9:20543581221106248. doi: 10.1177/20543581221106248 (PMC9218433; doi:10.1177/20543581221106248)
Supplement: sj-pdf-1-cjk-10.1177_20543581221106248 – Supplemental material for Impact of Home Telemonitoring and Management Support on Blood Pressure Control in Nondialysis CKD: A Systematic Review and Meta-Analysis [file sj-pdf-1-cjk-10.1177_20543581221106248.pdf]

SUPPLEMENTARY MATERIAL

Supplementary Table S1: Database search strategy

**MEDLINE search strategy: July 6, 2020, 188 results**		
1. exp Hypertension/<br>2. hypertens*.mp.<br>3. exp Blood Pressure/<br>4. blood pressure*.mp.<br>5. arter* pressure*.mp.<br>6. venous pressure*.mp.<br>7. vein pressure*.mp.<br>8. exp Blood Pressure Determination/<br>9. 1 or 2 or 3 or 4 or 5 or 6 or 7 or 8<br>10. exp Renal Insufficiency, Chronic/<br>11. Chronic Kidney disease*.mp.<br>12. chronic kidney insufficienc*.mp.<br>13. chronic renal disease*.mp.<br>14. chronic renal insufficienc*.mp.<br>15. CKD.mp.<br>16. Renal fail*.mp.<br>17. Kidney fail*.mp.<br>18. 10 or 11 or 12 or 13 or 14 or 15 or 16 or 17<br>19. exp Telemedicine/<br>20. telecare.mp.<br>21. telecollaborat*.mp.<br>22. teleconsult*.mp.<br>23. teleconference*.mp.<br>24. telehealth.mp.<br>25. teleguide*.mp.<br>26. telediagnos*.mp.	27. telemed*.mp.<br>28. telemonitor*.mp.<br>29. telepresence*.mp.<br>30. telerehab*.mp.<br>31. telerobotic*.mp.<br>32. telescreen*.mp.<br>33. teletransmi*.mp.<br>34. (consult* and (skype or facetime or internet or web based)).mp.<br>35. ((distan* or remote* or video* or virtual) adj2 (consult* or deliver* or diagnos* or monitor* or visit* or manag*)).mp.36. ehealth*.mp.<br>37. tele care.mp.<br>38. tele collaborat*.mp.<br>39. tele consult*.mp.<br>40. tele conference*.mp.<br>41. tele health.mp.<br>42. tele guide*.mp.<br>43. tele diagnos*.mp.<br>44. tele med*.mp.<br>45. tele monitor*.mp.<br>46. tele presence*.mp.<br>47. tele robotic*.mp.<br>48. tele screen*.mp.<br>49. tele transmi*.mp.	50. (teletherap* not (x-ray or radiat* or cobalt or gamma* or cesium)).mp.<br><br>51. telemetry/<br>52. telemetry.mp.<br>53. Telemetries.mp.<br>54. telenurs*.mp.<br>55. telephone/<br>56. Telephon*.mp.<br>57. smartphone/ - nt under mobile phone<br>58. smartphone*.mp.<br>59. Cell phone/<br>60. cellphone*.mp.<br>61. cell* phone*.mp.<br>62. internet/<br>63. internet*.mp.<br>64. (telenephrolog* or tele nephrolog*).mp.<br>65. Videoconferencing/<br>66. (videoconferenc* or video conferenc*).mp.<br>67. Mobile Applications/<br>68. mobile application*.mp.<br>69. or/19-68<br>70. 9 and 18 and 69
**Embase search strategy: 1974 to 2020 (July 2, 2020), 728 results**		
1. exp elevated blood pressure/<br>2. exp blood pressure/<br>3. exp blood pressure measurement/<br>4. hypertens*.mp.<br>5. blood pressure*.mp.<br>6. arter* pressure*.mp.<br>7. venous pressure*.mp.<br>8. vein pressure*.mp.<br>9. or/1-8<br>10. exp chronic kidney failure/<br>11. Chronic Kidney disease*.mp.<br>12. chronic kidney insufficienc*.mp.<br>13. chronic renal disease*.mp.<br>14. chronic renal insufficienc*.mp.<br>15. CKD.mp.<br>16. Renal fail*.mp.<br>17. Kidney fail*.mp.<br>18. or/10-17<br>19. exp telemedicine/<br>20. exp telemetry/<br>21. telephone/<br>22. exp mobile phone/<br>23. internet/<br>24. videoconferencing/<br>25. exp mobile application/<br>26. telecare.mp.	 27. telecollaborat*.mp.<br>28. teleconsult*.mp.<br>29. teleconference*.mp.30. telehealth.mp.<br><br>31. teleguide*.mp.<br>32. telediagnos*.mp.<br>33. telemed*.mp.<br>34. telemonitor*.mp.36. telerehab*.mp.<br>37. telerobotic*.mp.<br>38. telescreen*.mp.<br>39. teletransmi*.mp.<br>40. (consult* and (skype or facetime or internet or web based)).mp.<br>41. ((distan* or remote* or video* or virtual) adj2 (consult* or deliver* or diagnos* or monitor* or visit* or manag*)).mp.<br>42. ehealth*.mp.<br>43. tele care.mp.<br>44. tele collaborat*.mp.<br>45. tele consult*.mp.<br>46. tele conference*.mp.<br>47. tele health.mp.<br>48. tele guide*.mp.<br>49. tele diagnos*.mp.<br>50. tele med*.mp.	51. tele monitor*.mp.<br>52. tele presence*.mp.<br>53. tele robotic*.mp.<br>54. tele screen*.mp.<br>55. tele transmi*.mp.<br>56. (teletherap* not (x-ray or radiat* or cobalt or gamma* or cesium)).mp.<br><br>57. telemetry.mp.58. Telemetries.mp.<br><br>59. telenurs*.mp.<br>60. Telephon*.mp.<br>61. smartphone*.mp.<br>62. cellphone*.mp.<br>63. cell* phone*.mp.<br>64. internet*.mp.<br>65. (telenephrolog* or tele nephrolog*).mp.<br>66. (videoconferenc* or video conferenc*).mp.<br>67. mobile application*.mp.<br>68. or/19-67<br>69. 9 and 18 and 68
**APA PsycInfo: 1806 to June 2020 (June 30, 2020), 5 results**		

1. exp hypertension/<br>2. exp blood pressure/<br>3. hypertens*.mp.<br>4. blood pressure*.mp.<br>5. arter* pressure*.mp.<br>6. venous pressure*.mp.<br>7. vein pressure*.mp.<br>8. or/1-7<br>9. kidney diseases/<br>10. Chronic Kidney disease*.mp.<br>11. chronic kidney insufficienc*.mp.<br>12. chronic renal disease*.mp.<br>13. chronic renal insufficienc*.mp.<br>14. CKD.mp.<br>15. Renal fail*.mp.<br>16. Kidney fail*.mp.<br>17. or/9-16<br>18. exp Telemedicine/<br>19. telemetry/<br>20. exp telephone systems/<br>21. internet/<br>22. videoconferencing/<br>23. mobile applications/<br>24. telecare.mp.<br>25. telecollaborat*.mp.	26. teleconsult*.mp.27. teleconference*.mp.<br>28. telehealth.mp.<br>29. teleguide*.mp.<br>30. telediagnos*.mp.<br>31. telemed*.mp.<br>32. telemonitor*.mp.<br>33. telepresence*.mp.<br>34. telerehab*.mp.<br>35. telerobotic*.mp.<br>36. telescreen*.mp.<br>37. teletransmi*.mp.<br>38. (consult* and (skype or facetime or internet or web based)).mp.<br>39. ((distan* or remote* or video* or virtual) adj2 (consult* or deliver* or diagnos* or monitor* or visit* or manag*)).mp.<br>40. ehealth*.mp.<br>41. tele care.mp.<br>42. tele collaborat*.mp.<br>43. tele consult*.mp.<br>44. tele conference*.mp.<br>45. tele health.mp.<br>46. tele guide*.mp.	47. tele diagnos*.mp.<br>48. tele med*.mp.<br>49. tele monitor*.mp.50. tele presence*.mp.<br>51. tele robotic*.mp.<br>52. tele screen*.mp.<br>53. tele transmi*.mp.<br>54. (teletherap* not (x-ray or radiat* or cobalt or gamma* or cesium)).mp.<br><br>55. telemetry.mp.<br>56. Telemetries.mp.<br>57. telenurs*.mp.<br>58. Telephon*.mp.<br>59. smartphone*.mp.<br>60. cellphone*.mp.<br>61. cell* phone*.mp.<br>62. internet*.mp.<br>63. (telenephrolog* or tele nephrolog*).mp.<br>64. (videoconferenc* or video conferenc*).mp.<br>65. mobile application*.mp.<br>66. or/18-65<br>67. 8 and 17 and 66
**CINAHL Plus with full text (EBSCO): 111 results**		
S1 (MH "Hypertension+")<br>S2 (MH "Blood Pressure+")<br>S3 (MH "Blood Pressure Determination")<br>S4 hypertens*<br>S5 "blood pressure*"<br>S6 "arter* pressure*"<br>S7 "venous pressure*"<br>S8 "vein pressure*"<br>S9 S1 OR S2 OR S3 OR S4 OR S5 OR S6 OR S7 OR S8<br>S10 (MH "Kidney Failure, Chronic+")<br>S11 "Chronic Kidney disease*"<br>S12 "chronic kidney insufficienc*"<br>S13 "chronic renal disease*"<br>S14 "chronic renal insufficienc*"<br>S15 CKD<br>S16 "Renal fail*"<br>S17 "Kidney fail*"<br>S18 S10 OR S11 OR S12 OR S13 OR S14 OR S15 OR S16 OR S17<br>S19 (MH "Telemedicine+")<br>S20 (MH "Telemetry")<br>S21 (MH "Telephone+")<br>S22 (MH "Internet")<br>S23 (MH "Mobile Applications")<br>S24 telecare<br>S25 telecollaborat*<br>S26 teleconsult*	S27 teleconference*<br>S28 telehealth<br>S29 teleguide*<br>S30 telediagnos*<br>S31 telemed*<br>S32 telemonitor*<br>S33 telepresence*<br>S34 telerehab*<br>S35 telerobotic*<br>S36 telescreen*<br>S37 teletransmi*<br>S38 (consult* AND (skype OR facetime OR internet OR "web based"))<br>S39 ((distan* OR remote* OR video* OR virtual) N2 (consult* OR deliver* OR diagnos* OR monitor* OR visit* OR manag*))<br>S40 ehealth*<br>S41 "tele care"<br>S42 "tele collaborat*"<br>S43 "tele consult*"<br>S44 "tele conference*"<br>S45 "tele health"<br>S46 "tele guide*"<br>S47 "tele guide*"<br>S48 "tele diagnos*"<br>S49 "tele med*"<br>S50 "tele monitor*"<br>S51 "tele presence*"<br>S52 "tele robotic*"<br>S53 "tele robotic*"	S54 "tele screen*"<br>S55 "tele transmi*"<br>S56 (teletherap* NOT ("x-ray" OR radiat* OR cobalt OR gamma* OR cesium))<br>S57 telemetry<br>S58 Telemetries<br>S59 telenurs*<br>S60 Telephon*<br>S61 smartphone*<br>S62 cellphone*<br>S63 "cell* phone*"<br>S64 internet*<br>S65 (telenephrolog* OR "tele nephrolog*")<br>S66 (videoconferenc* OR "video conferenc*")<br>S67 "mobile application*"<br>S68 S19 OR S20 OR S21 OR S22 OR S23 OR S24 OR S25 OR S26 OR S27 OR S28 OR S29 OR S30 OR S31 OR S32 OR S33 OR S34 OR S35 OR S36 OR S37 OR S38 OR S39 OR S40 OR S41 OR S42 OR S43 OR S44 OR S45 OR S46 OR S47 OR S48 OR S49 OR S50 OR S51 OR S52 OR S53 OR S54 OR S55 OR S56 OR S57 OR S58 OR S59 OR S60 OR S61 OR S62 OR S63 OR S64 OR S65 OR S66 OR S67<br>S69 S9 AND S18 AND S68
**Cochrane CENTRAL: 449 results**		
#1 MeSH descriptor: [Hypertension] explode all trees<br>#2 hypertens*	#23 teleconference*<br>#24 telehealth<br>#25 teleguide*	#49 tele NEXT transmi*<br>#50 teletherap* NOT ("x-ray" OR radiat* OR cobalt OR gamma*

#3 MeSH descriptor: [Blood Pressure] explode all trees<br>#4 MeSH descriptor: [Blood Pressure Determination] explode all trees<br>#5 arter* NEXT pressure*<br>#6 blood NEXT pressure*<br>#7 venous NEXT pressure*<br>#8 vein NEXT pressure*<br>#9 {OR #1-#8}<br>#10 MeSH descriptor: [Renal Insufficiency, Chronic] explode all trees<br>#11 "Chronic Kidney" NEXT disease*<br>#12 "chronic kidney" NEXT insufficienc*<br>#13 "chronic renal" NEXT disease*<br>#14 "chronic renal" NEXT insufficienc*<br>#15 CKD<br>#16 Renal NEXT fail*<br>#17 Kidney NEXT fail*<br>#18 {OR #10-#17}<br>#19 MeSH descriptor: [Telemedicine] explode all trees<br>#20 telecare<br>#21 telecollaborat*<br>#22 teleconsult*	#26 telediagnos*<br>#27 telemed*<br>#28 telemonitor*<br>#29 telepresence*<br>#30 telerehab*<br>#31 telerobotic*<br>#32 telescreen*<br>#33 teletransmi*<br>#34 consult* AND (skype OR facetime OR internet OR "web based")<br>#35 (distan* OR remote* OR video* OR virtual) NEXT (consult* OR deliver* OR diagnos* OR monitor* OR visit* OR manag*)<br>#36 ehealth*<br>#37 "tele care"<br>#38 tele NEXT collaborat*<br>#39 tele NEXT consult*<br>#40 tele NEXT conference*<br>#41 "tele health"<br>#42 tele NEXT guide*<br>#43 tele NEXT diagnos*<br>#44 tele NEXT med*<br>#45 tele NEXT monitor*<br>#46 tele NEXT presence*<br>#47 tele NEXT robotic*<br>#48 tele NEXT screen*	OR cesium)#51 MeSH descriptor: [Telemetry] this term only<br>#52 telemetry<br>#53 Telemetries<br>#54 telenurs*<br>#55 MeSH descriptor: [Telephone] this term only<br>#56 Telephon*<br>#57 MeSH descriptor: [Smartphone] this term only<br>#58 smartphone*<br>#59 MeSH descriptor: [Cell Phone] this term only<br>#60 cellphone*<br>#61 cell NEXT phone*<br>#62 MeSH descriptor: [Internet] this term only<br>#63 internet*<br>#64 telenephrolog*<br>#65 tele NEXT nephrolog*<br>#66 MeSH descriptor: [Videoconferencing] this term only<br>#67 videoconferenc*<br>#68 video NEXT conferenc*<br>#69 MeSH descriptor: [Mobile Applications] this term only<br>#70 mobile NEXT application*<br>#71 {OR #19-#70}<br>#72 #9 AND #18 AND #71

WoS Core Collection: 178 results (Indexes: SCI-EXPANDED, SSCI, A&HCI, CPCI-S, CPCI-SSH, BKCI-S, BKCI-SSH, ESCI, CCR-EXPANDED, IC)

TOPIC: (((hypertens* OR "Blood pressure*" OR "ater* pressure*" OR "vein pressure*" OR "venous pressure*"))) AND TOPIC: (("Chronic Kidney disease*" OR "chronic kidney insufficienc*" OR "chronic renal disease*" OR "chronic renal insufficienc*" OR "CKD" OR "Renal fail*" OR "Kidney fail*")) AND TOPIC: ((Telecare OR telecollaborat* OR teleconsult* OR teleconference* OR telehealth OR teleguide* OR telediagnos* OR telemed* OR telemonitor* OR telepresence* OR telerehab* OR telerobotic* OR telescreen* OR teletransmi* OR (consult* AND (skype OR facetime OR internet OR "web based")) OR ((distan* OR remote* OR video* OR virtual) NEAR/2 (consult* OR deliver* OR diagnos* OR monitor* OR visit* OR manag*)) OR ehealth* OR "tele care" OR "tele collaborat*" OR "tele consult*" OR "tele conference*" OR "tele health" OR "tele guide*" OR "tele diagnos*" OR "tele med*" OR "tele monitor*" OR "tele presence*" OR "tele robotic*" OR "tele screen*" OR "tele transmi*" OR (teletherap* NOT ("x-ray" OR radiat* OR cobalt OR gamma* OR cesium)) OR telemetry OR telemetries OR telenurs* OR Telephon* OR smartphone* OR cellphone* OR "cell* phone*" OR internet* OR telenephrolog* or "tele nephrolog*" OR videoconferenc* OR "video conferenc*" OR "mobile application*"))

Dissertations & Theses Global (ProQuest): 5 results

noft((hypertens* OR "Blood pressure*" OR "ater* pressure*" OR "vein pressure*" OR "venous pressure*")) AND noft("Chronic Kidney disease*" OR "chronic kidney insufficienc*" OR "chronic renal disease*" OR "chronic renal insufficienc*" OR "CKD" OR "Renal fail*" OR "Kidney fail*") AND noft(Telecare OR telecollaborat* OR teleconsult* OR teleconference* OR telehealth OR teleguide* OR telediagnos* OR telemed* OR telemonitor* OR telepresence* OR telerehab* OR telerobotic* OR telescreen* OR teletransmi* OR (consult* AND (skype OR facetime OR internet OR "web based")) OR ((distan* OR remote* OR video* OR virtual) NEAR/2 (consult* OR deliver* OR diagnos* OR monitor* OR visit* OR manag*)) OR ehealth* OR "tele care" OR "tele collaborat*" OR "tele consult*" OR "tele conference*" OR "tele health" OR "tele guide*" OR "tele diagnos*" OR "tele med*" OR "tele monitor*" OR "tele presence*" OR "tele robotic*" OR "tele screen*" OR "tele transmi*" OR (teletherap* NOT ("x-ray" OR radiat* OR cobalt OR gamma* OR cesium)) OR telemetry OR telemetries OR telenurs* OR Telephon* OR smartphone* OR cellphone* OR "cell* phone*" OR internet* OR telenephrolog* or "tele nephrolog*" OR videoconferenc* OR "video conferenc*" OR "mobile application*")

Supplementary Table S2: Quality assessment checklist for prevalence studies

**Name of author(s):**		
**Year of publication:**		
**Study title:**		
**Risk of bias items**	**Risk of bias levels**	**Points scored**
**1.** Was the study's target population a close representation of the national population in relation to relevant variables, e.g. age, sex, occupation?	**Yes** (**LOW RISK**): The study's target population was a close representation of the national population.	
	**No** (**HIGH RISK**): The study's target population was clearly NOT representative of the national population.	
**2.** Was the sampling frame a true or close representation of the target population?	**Yes** (**LOW RISK**): The sampling frame was a true or close representation of the target population.	
	**No** (**HIGH RISK**): The sampling frame was NOT a true or close representation of the target population.	
**3.** Was some form of random selection used to select the sample, OR, was a census undertaken?	**Yes** (**LOW RISK**): A census was undertaken, OR, some form of random selection was used to select the sample (e.g. simple random sampling, stratified random sampling, cluster sampling, systematic sampling).	
	**No** (**HIGH RISK**): A census was NOT undertaken, AND some form of random selection was NOT used to select the sample	
**4.** Was the likelihood of non-response bias minimal?	**Yes** (**LOW RISK**): The response rate for the study was ≥75%, OR, an analysis was performed that showed no significant difference in relevant demographic characteristics between responders and non- responders	
	**No** (**HIGH RISK**): The response rate was <75%, and if any analysis comparing responders and non-responders was done, it showed a significant difference in relevant demographic characteristics between responders and non-responders	
**5.** Were data collected directly from the subjects (as opposed to a proxy)?	**Yes** (**LOW RISK**): All data were collected directly from the subjects.	
	**No** (**HIGH RISK**): In some instances, data were collected from a proxy.	
**6.** Was an acceptable case definition used in the study?	**Yes** (**LOW RISK**): An acceptable case definition was used.	
	**No** (**HIGH RISK**): An acceptable case definition was NOT used	
**7.** Was the study instrument that measured the parameter of interest (e.g. prevalence of low back pain) shown to have reliability and validity (if necessary)?	**Yes** (**LOW RISK**): The study instrument had been shown to have reliability and validity (if this was necessary), e.g. test-re- test, piloting, validation in a previous study, etc.	
	**No** (**HIGH RISK**): The study instrument had NOT been shown to have reliability or validity (if this was necessary).	
**8.** Was the same mode of data collection used for all subjects?	**Yes** (**LOW RISK**): The same mode of data collection was used for all subjects.	
	**No** (**HIGH RISK**): The same mode of data collection was NOT used for all subjects.	
**9.** Were the numerator(s) and denominator(s) for the parameter of interest appropriate	**Yes** (**LOW RISK**): The paper presented appropriate numerator(s) AND denominator(s) for the parameter of interest (e.g. the prevalence of low back pain).	
	**No** (**HIGH RISK**): The paper did present numerator(s) AND denominator(s) for the parameter of interest but one or more of these were inappropriate.	
**10.** Summary on the overall risk of study bias	**LOW RISK**	0 – 3
	**MODERATE RISK**	4 – 6
	**HIGH RISK**	7 – 9

Hoy D, Brooks P, Woolf A, Blyth F, March L, Bain C, et al. Assessing risk of bias in prevalence studies: modification of an existing tool and evidence of interrater agreement. J Clin Epidemiol. 2012;65: 934-939.

Supplementary Figure S1: Baseline assessment for SBP, DBP, and eGFR.

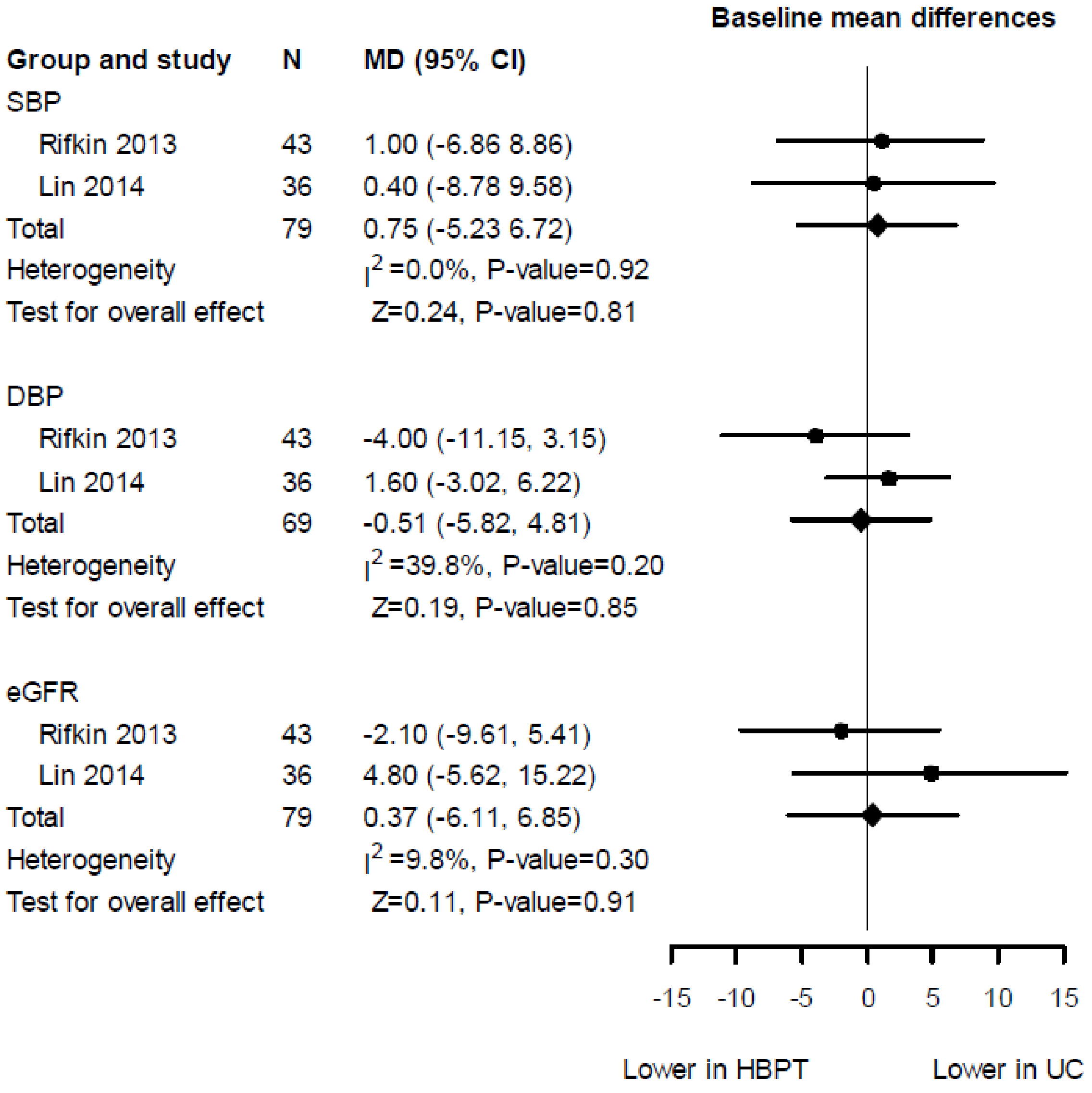

MD is calculated as baseline mean from HBPT minus baseline mean from UC.

Abbreviations: SBP – systolic blood pressure; DBP – diastolic blood pressure; eGFR – estimated glomerular filtration rate; MD – mean difference; CI – confidence interval.; HBPT – home blood pressure telemonitoring; UC – usual care.

Supplementary Figure S2: Forest plot and meta-analysis for the effect of HBPT on SBP and DBP after 3 months of follow-up.

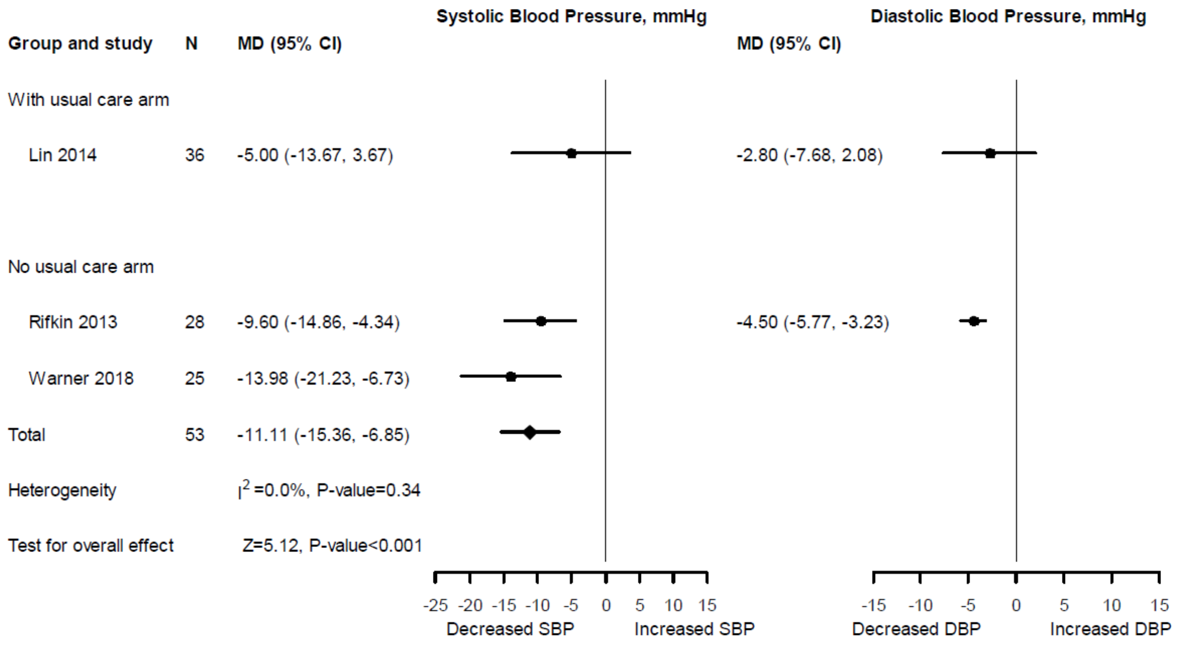

The usual care arm group includes studies with both HBPT and UC arms; MD is calculated as the difference between HBPT and UC in the mean changes from baseline (i.e., follow-up minus baseline values). The no usual care arm group includes studies with an HBPT arm only; MD is calculated as the follow-up mean minus the baseline mean.

Abbreviations: HBPT – home blood pressure telemonitoring; SBP – systolic blood pressure; DBP – diastolic blood pressure; MD – mean difference; CI – confidence interval.

SUPPLEMENTARY APPENDIX

Knowledge Translation Process

Project Title: Impact of Home Telemonitoring and Management Support on Blood Pressure Control in Non-dialysis CKD: A Systematic Review Protocol

The results of this review will be delivered to knowledge users (KUs) to be implemented into healthcare models to improve blood pressure (BP) control in a home-based setting. This is important for optimal control and management of hypertension, as it is a high risk factor for CKD. We will do this utilizing the following end-of-grant knowledge translation (KT) plan:

End-of-grant KT plan:[1, 2]

(i) ***Objective:*** We plan to use the information obtained from the systematic review to generate awareness about the impacts of HBPT programs on CKD and CVD, and to share this knowledge to inform policies and guidelines to improve healthcare delivery. We aim to communicate the results of the study in an interpretable and easily accessible manner to help the target audience incorporate the results in clinical practice and modify recommendations, if applicable.

(ii) ***Target audience:*** Our target audience is KUs, including: clinicians and researchers with expertise in renal disease, internal medicine, cardiovascular disease, knowledge translation, epidemiology and biostatistics; patients; policy makers; funding agencies; and members of the Public Health Agency of Canada, Canadian Cardiovascular Society, Hypertension Canada, Heart and Stroke Foundation of Canada, Strategy for Patient-Oriented Research (SPOR) support units in Alberta and Canada, and the Canadian Society of Nephrology **(Table 1)**.

(iii) ***Methods and tools:*** We will use active and passive methods to share our study findings[3] [4]. Passive methods include sending infographics (or graphical abstracts) to KUs and publishing articles in peer-reviewed journals. Active methods include conducting a needs assessment with KUs, tailoring the results to help them implement the findings into practice, and sharing these insights in conference presentations, reports and policy briefs.

(iv) ***Timing:*** The results will be synthesized at the end of the study.

(v) ***Responsibility:*** The research team will work with the Alberta SPOR Unit, project partners and KUs for dissemination.

Additional details about the end-of-grant KT plan can be found in Table 2 of this appendix.

Knowledge Exchange Process:

We will contact KUs in multiple ways including email, phone and social media to inform them about the project and ask for their support. Dialogues and discussions between the research team and KUs will determine the extent of each KU's involvement. Involvement in the project may motivate KUs to implement evidence about the potential benefits of HBPT. We will engage them in multiple stages of the project, including data tabulation, knowledge synthesis and dissemination, and will incorporate their feedback. This will ensure that all KUs will be able to easily understand the final results and overcome potential barriers, such as geographic differences in patterns of clinical practice. Challenges for KT include language and health evidence not being aligned with KU needs. A major challenge is translating evidence into a KT policy that meets each KU's specific needs. To overcome these potential barriers, the results will be shared and discussed with KUs, who will help synthesize them to ensure the information is easily understood. The study team will work with the KUs to help translate the health evidence in accordance with linguistic and end-user needs, thereby ensuring the results are delivered in a format that is applicable and beneficial for end-users.

Table 1: Integrated KT Plan (based on the CIHR integrated KT (iKT) plan,[2])

Task	Knowledge users/research team	Notes
Collectively make decisions to shape the research questions	Nephrologists	Dr. Aminu Bello and Dr. Ikechi Okpechi
Collectively interpret the findings from the study	Clinicians and researchers from the University of Alberta, University of Calgary and Alberta Health Services with expertise in renal disease, internal medicine, cardiovascular disease, knowledge translation, epidemiology and biostatistics; patients; policy makers from the governments of Alberta and Canada; funding agencies such as the Canadian Institutes of Health Research; members of the Public Health Agency of Canada, the Canadian Cardiovascular Society, Hypertension Canada, the Heart and Stroke Foundation of Canada, Strategy for Patient-Oriented Research (SPOR) support units in Alberta and Canada, and the Canadian Society of Nephrology	The research team will work with knowledge users to interpret the data and share study findings
Craft messages around the results and incorporate them into practice	Clinicians and researchers from the University of Alberta, University of Calgary and Alberta Health Services with expertise in renal disease, internal medicine, cardiovascular disease, knowledge translation, epidemiology and biostatistics; patients; policy makers from the governments of Alberta and Canada; funding agencies such as the Canadian Institutes of Health Research; members of the Public Health Agency of Canada, the Canadian Cardiovascular Society, Hypertension Canada, the Heart and Stroke Foundation of Canada, Strategy for Patient-	Synthesize information about the positive impacts of HBPT on CKD and CVD related health outcomes, share knowledge to inform decision-making and potential changes to relevant health policies

Oriented Research (SPOR) support units in Alberta and Canada, and the Canadian Society of Nephrology

Table 2: End-of-grant KT Plan:[1]

Knowledge users and audiences	KT goals	Main messages	KT strategies	Evaluation	Resources
Clinicians and researchers from the University of Alberta, University of Calgary and Alberta Health Services with expertise in renal disease, internal medicine, cardiovascular disease, knowledge translation, epidemiology and biostatistics; patients; policy makers from the governments of Alberta and Canada; funding agencies such as the Canadian Institutes of Health Research; members of the Public Health Agency of Canada, Canadian Cardiovascular Society, Hypertension Canada, Heart and Stroke Foundation of Canada, Strategy for Patient-Oriented Research (SPOR) support units in Alberta and Canada, and the Canadian Society of Nephrology	Synthesize information about the positive impacts of HBPT on CKD- and CVD- related health outcomes, share knowledge to inform decision-making and potential changes to relevant health policies	How HBPT impacts blood pressure control and health outcomes associated with cardiovascular disease and kidney disease	Publications and infographics about HBPT<br><br>Presentation of results at conferences<br><br>Policy briefs	Reach indicator: Number of KUs who receive study results and policy briefs<br><br>Use indicator: Number of KUs who use study data to adapt health policies and improve clinical practice guidelines and research<br><br>Policy indicator: Changes implemented in policy briefs based on the results of the study	Time for meetings (virtual and in-person)<br><br>Financial resources for publications, infographics, and posters for presentations at conferences and workshops

Feasibility:

Knowledge users will include researchers and clinicians with expertise in internal medicine, kidney disease, cardiovascular disease, biostatistics, epidemiology and knowledge translation; patients; decision makers; policy makers/government agencies; funding agencies for research; members of the Public Health Agency of Canada, Hypertension Canada, the Canadian Cardiovascular Society, the Heart and Stroke Foundation of Canada, the Canadian Society of Nephrology, the International Society of Nephrology, Kidney Disease: Improving Global Outcomes (KDIGO), and Strategy for Patient-Oriented Research (SPOR) support units for primary care in Alberta and across Canada.

Anticipated outcomes:

1. ***Improved healthcare delivery:*** The study will summarize outcomes of HBPT and effects on CKD- and CVD-related health outcomes. This information will help clinicians and health systems implement the HBPT intervention in a timely fashion.
2. ***Optimal care for patients:*** Results obtained through this systematic review facilitate the implementation of successful HBPT strategies in various communities. This will be vector for optimal care delivery, as it may lead to better BP control in patients.
3. ***Potential reduction in unnecessary healthcare costs:*** Provision of effective care will help save costs related to poor BP control, such as costs for medications, hospitalizations related to complications of uncontrolled hypertension, and kidney replacement therapy.

References:

1. Research CIoH: **A Guide to Researcher and Knowledge-User Collaboration in Health Research**. *https://cihr-irscgcca/e/44954html*.
2. Canadian I, of, Health, Research: **Guide to knowledge translation planning at CIHR: Integrated and end-of-grant approaches**: Canadian Institutes of Health Research; 2012.
3. Majumdar SR SS: **Why most interventions to improve physician prescribing do not seem to work.** *CMAJ : Canadian Medical Association journal = journal de l'Association medicale canadienne* 2003, **Jul 8;169(1):30-1**.
4. Grimshaw JM SL, Thomas R, et al.: **Changing provider behavior: an overview of systematic reviews of interventions.** *Med Care 2001 Aug;39(8 Suppl 2):II2-45* 2001.
